# Supplementary material for: Effectiveness of Protein Supplementation Combined with Resistance Training on Muscle Strength and Physical Performance in Elderly: A Systematic Review and Meta-Analysis
Source: Nutrients. 2020 Aug 27;12(9):2607. doi: 10.3390/nu12092607 (PMC7551830; doi:10.3390/nu12092607)
Supplement: Supplementary file 1 [file nutrients-12-02607-s001.zip › supplementary/Supplementary S1. Complete literature search.docx]

**Supplementary S1:** Complete literature search

**PubMed Search Formula**

#1 (aged [mh] OR aged OR "old people" OR "older people" OR "older adults" OR "old adults" OR elderly OR senior OR geriatric OR frail)

#2 (resistance training [mh] OR resistance training OR strength training)

#3 ("protein supplementation" OR "supplemental protein")

#4 (physical fitness [mh] OR physical fitness OR functionality OR Performance OR Strength OR Resistance OR Endurance OR balance OR stability OR agility OR mobility OR Gait OR Speed OR Locomotion OR fall OR Handgrip OR SPPB OR Tandem OR TUG OR "Timed up and go" OR "quality of life")

#5 (randomized controlled trial [pt] OR controlled clinical trial [pt] OR randomized [TiAb]) OR placebo [TiAb] OR clinical trials as topic [mesh: noexp] OR randomly [TiAb]) OR trial [Ti]) NOT (animals [mh] NOT humans [mh])

#6 #1 AND #2 AND #3 AND #4 AND #5

Results: 95

Date: 18/05/2020

**Cochrane Library Search Formula**

#1 MeSH descriptor: [Aged] explode all trees

#2 aged OR "old people" OR "older people" OR "older adults" OR "old adults" OR elderly OR senior OR geriatric OR frail

#3 #1 OR #2

#4 "resistance training" OR "strength training"

#5 "protein supplementation" OR "supplemental protein"

#6 MeSH descriptor: [Physical Fitness] explode all trees

#7 physical fitness OR functionality OR Performance OR Strength OR Resistance OR Endurance OR balance OR stability OR agility OR mobility OR Gait OR Speed OR Locomotion OR fall OR Handgrip OR SPPB OR Tandem OR TUG OR "Timed up and go" OR "quality of life"

#8 #6 OR #7

#9 #3 AND #4 AND #5 AND #8

Results: 105

Date: 18/05/2020

**Web of Science Search Formula**

#1 ALL=(aged OR “old people” OR “older people” OR “older adults” OR “old adults” OR elderly OR senior OR geriatric OR frail)

#2 ALL=(“resistance training” OR “strength training”)

#3 ALL=(“protein supplementation” OR supplemental protein”

#4 ALL=(physical fitness OR functionality OR Performance OR Strength OR Resistance OR Endurance OR balance OR stability OR agility OR mobility OR Gait OR Speed OR Locomotion OR fall OR Handgrip OR SPPB OR Tandem OR TUG OR "Timed up and go" OR "quality of life")

#5 TS=research design OR TS=comparative stud* OR TS=evaluation stud* OR TS=controlled trial* OR TS=follow-up stud* OR TS=prospective stud* OR TS=random* OR TS=placebo* OR TS=(single blind*) OR TS=(double blind*)

#6 (TS=#1 AND #2 AND #3 AND #4 AND #5)

Results: 94

Date: 18/05/2020
