# Supplementary material for: Effectiveness of Protein Supplementation Combined with Resistance Training on Muscle Strength and Physical Performance in Elderly: A Systematic Review and Meta-Analysis
Source: Nutrients. 2020 Aug 27;12(9):2607. doi: 10.3390/nu12092607 (PMC7551830; doi:10.3390/nu12092607)
Supplement: Supplementary file 1 [file nutrients-12-02607-s001.zip › supplementary/Supplementary S2. Additional characteristics of the resistance training protocols.docx]

| **Supplementary S2. Additional characteristics of the resistance training protocols** | | | | | | | | | |
| --- | --- | --- | --- | --- | --- | --- | --- | --- | --- |
| **Study** | **Type of exercise** | **Volume load progression*** | | **Number of exercises** | **Single joint-multi joint exercises** | | **Mean rate of volume load increase** | **Proximity to failure** | **Volume split/body part** |
|  |  | **Baseline volume load** | **Final volume load** |  | |  |  |  |  |
| Amasene, 2019 [31] | UB and LB resistance training + dynamic balance exercises | 96 x NS x 50-65 | 168 x NS x 70 | 6 | | 4-2 | NS | NS | NS |
| Arnarson, 2013 [30] | UB and LB resistance training | NS x NS x 60 | 990 x 6-8 x 75-80 | 10 | | 7-3 | 5-10%/week | 6-8 RM | NS |
| Candow 2008 [32] | UB and LB resistance training | NS | 810 x 10 x 70 | 9 | | 5-4 | NS | 10-RM | NS |
| Holwerda, 2018 [33] | UB and LB resistance training | 144 x 8-10 x 70-80 | 288 x 10 x 80 | 6 | | 1-5 | NS | 10-RM | NS |
| Krause, 2019 [34] | UB and LB bodyweight and elastic bands resistance training | 270 x 8-12 x NS | 990 x 10-14 x NS | 10 | | 3-7 | NS | NS | NS |
| Leenders, 2013 [35] | UB and LB resistance training | 216 x 8-15 x 60-75 | 1200 x 8 x 75-80 | 8 | | 3-5 | NS | 8-RM | LB exercises:  480 x 8 x 75-80  UB exercises:  720 x 8 x 75-80 |
| Mori, 2018 [36] | UB and LB bodyweight and elastic bands resistance training | NS | NS x NS x 50-70 | 7 | | 2-5 | NS | NS | NS |
| Nabuco, 2018 [37] | UB and LB resistance training | 576 x 10 x NS | 576 x 8-12 x NS | 8 | | 5-3 | NS | 8-12 RM | NS |
| Nabuco, 2019 [38] | UB and LB resistance training | 576 x 10 x NS | 576 x 8-12 x NS | 8 | | 5-3 | 2 to 5 % for UB exercises  5 to 10% for LB exercises | 8-12 RM | NS |
| Stragier S, 2016 [39] | UB and LB resistance training | NS x 10 x NS | NS x 10 x NS | NS | | NS | NS | 10 RM | NS |
| Sugihara, 2018 [40] | UB and LB resistance training | 576 x 8-12 x NS | 864 x 8-12 x NS | 8 | | 5-3 | 2 to 5 % for UB exercises  5 to 10% for LB exercises | 8-12 RM | NS |
| Tieland, 2012 [41] | UB and LB resistance training | NS x 10-15 x 50 | NS x 8-10 x 75 | 6 | | 1-5 | NS | NS | NS |
| Trabal, 2015 [29] | UB and LB resistance training + balance exercises | NS | 324 x 8 x NS | 9 | | 6-3 | NS | NS | NS |
| Verdijk, 2009 [42] | LB resistance training | 96 x 8-15 x 60-75 | 96 x 8 x 75-80 | 2 | | 1-1 | NS | 8 RM | NS |
| Villanueva, 2014 [43] | UB and LB resistance training | NS | NS x 3-12 x 70 | 7 | | 1-6 | NS | Close but not to failure | NS |
| Zdzieblik D, 2015 [44] | UB and LB resistance training | NS | NS | NS | | NS | NS | NS | NS |
| * Volume load expressed as number of sets x number of repetitions x %1RM.  NS: not specified; RM: repetition maximum; LB: lower-body; UB: upper-body | | | | | | | | | |
