# Supplementary material for: Effectiveness of Protein Supplementation Combined with Resistance Training on Muscle Strength and Physical Performance in Elderly: A Systematic Review and Meta-Analysis
Source: Nutrients. 2020 Aug 27;12(9):2607. doi: 10.3390/nu12092607 (PMC7551830; doi:10.3390/nu12092607)
Supplement: Supplementary file 1 [file nutrients-12-02607-s001.zip › supplementary/Supplementary S3. Additional sample characteristics.docx]

| **Supplementary S3. Additional sample characteristics.** | | | | | |
| --- | --- | --- | --- | --- | --- |
| **Study** | **Eating habits of the participants** | | **Overall protein intake** | | **Proportion of sarcopenic individuals** |
|  | **Protein group** | **Placebo group** | **Protein group** | **Placebo group** |  |
| Amasene, 2019 [31] | 0% malnourished and 27% at risk of malnutrition according to MNA scores | 7.7% malnourished and 61.5% at risk of malnutrition according to MNA scores | NS | NS | All sarcopenic |
| Arnarson, 2013 [30] | NS | NS | Baseline: 1.00±0.3 g/kg/day  Endpoint: 1.06±0.2 g/kg/day | Baseline: 0.92±0.3 g/kg/day  Endpoint: 0.89±0.2 g/kg/day | NS |
| Candow 2008 [32] | No difference between groups following the Interactive Healthy Eating Index | | Baseline: 103±9 g/day  Endpoint: 104±9 g/day | Baseline: 92±6 g/day  Endpoint: 101±9 g/day | Healthy |
| Holwerda, 2018 [33] | Significant increase in total energy intake, Vitamin D and carbohydrate intake pre-post intervention. | Significant increase in total energy intake and carbohydrate intake pre-post intervention. | Baseline: 87±3 g/day  Endpoint: 111±3 g/day | Baseline: 93±4 g/day  Endpoint: 94±4 g/day | Healthy |
| Krause, 2019 [34] | NS | NS | NS | NS | Healthy |
| Leenders, 2013 [35] | No difference between groups in total energy intake or macronutrient composition. | | Men:  Baseline: 1.1±0.1 g/kg/day  Endpoint: 1.0±0.1 g/kg/day  Women:  Baseline: 1.2±0.1 g/kg/day  Endpoint: 1.2±0.1 g/kg/day | | Healthy |
| Mori, 2018 [36] | No difference between groups in total energy intake or macronutrient composition. | | Baseline: 1.3±0.0 g/kg/day  Endpoint: 1.4±0.0 g/kg/day | Baseline: 1.3±0.0 g/kg/day  Endpoint: 1.4±0.1 g/kg/day | NS |
| Nabuco, 2018 [37] | No difference between groups in total energy intake or macronutrient composition. | | Baseline: 0.9±0.2 g/kg/day  Endpoint: 1.4±0.3 g/kg/day | Baseline: 0.95±0.3 g/kg/day  Endpoint: 1±0.3 g/kg/day | NS |
| Nabuco, 2019 [38] | No difference between groups in total energy intake or macronutrient composition. | | (Without supplementation)  Baseline: 0.9±0.4 g/kg/day  Endpoint: 1±0.2 g/kg/day | (Without supplementation)  Baseline: 1.0±0.3 g/kg/day  Endpoint: 1.0±0.2 g/kg/day | All sarcopenic and obese |
| Stragier S, 2016 [39] | NS | | Baseline: NS  Endpoint: 1.2±0.3 g/kg/day | Baseline: NS  Endpoint: 1.1±0.3 g/kg/day | Healthy |
| Sugihara, 2018 [40] | Significantly higher total energy intake pre-post intervention. | Significantly higher carbohydrate, protein and total energy intake pre-post intervention | Baseline: 0.9±0.1 g/kg/day  Endpoint: 1.4±0.1 g/kg/day | Baseline: 0.8±0.1 g/kg/day  Endpoint: 0.9±0.1 g/kg/day | NS |
| Tieland, 2012 [41] | No difference between groups in total energy intake or macronutrient composition. | | Baseline: 1.0±0.1 g/kg/day  Endpoint: 1.3±0.3 g/kg/day | Baseline: 1.0±0.1 g/kg/day  Endpoint: 0.9±0.1 g/kg/day | Healthy but frail |
| Trabal, 2015 [29] | No difference between groups in total energy intake or macronutrient composition. | | Baseline: 1.3 g/kg/day  4-week follow-up: 1.3 g/kg/day  Endpoint: NS | Baseline: 1.2 g/kg/day  4-week follow-up: 1.4 g/kg/day  Endpoint: NS | NS |
| Verdijk, 2009 [42] | No difference between groups in total energy intake or macronutrient composition. | | (Without supplementation)  Baseline: 1.1±0.1 g/kg/day  Endpoint: 1.1±0.1 g/kg/day | (Without supplementation)  Baseline: 1.1±0.1 g/kg/day  Endpoint: 1.1±0.1 g/kg/day | Healthy |
| Villanueva, 2014 [43] | No difference between groups in total energy intake or macronutrient composition. | | NS | NS | Healthy |
| Zdzieblik D, 2015 [44] | No difference between groups in total energy intake or macronutrient composition. | | NS | NS | Sarcopenic |
| MNA: mini nutritional assessment; NS: non specified. | | | |  |  |
