# Supplementary material for: Effectiveness of Protein Supplementation Combined with Resistance Training on Muscle Strength and Physical Performance in Elderly: A Systematic Review and Meta-Analysis
Source: Nutrients. 2020 Aug 27;12(9):2607. doi: 10.3390/nu12092607 (PMC7551830; doi:10.3390/nu12092607)
Supplement: Supplementary file 1 [file nutrients-12-02607-s001.zip › supplementary/Supplementary S4. Subgroup Analysis by Sarcopenia Condition.docx]

**Supplementary S4:** Subgroup Analysis by Sarcopenia Condition


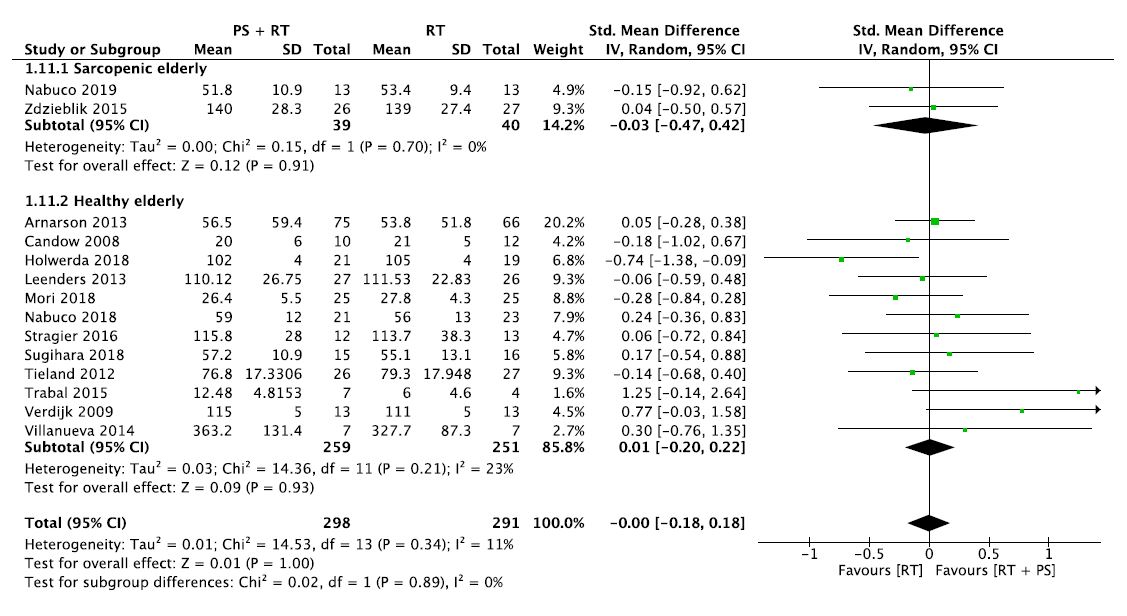


**Figure 1:** EEII Strength by Sarcopenia Condition


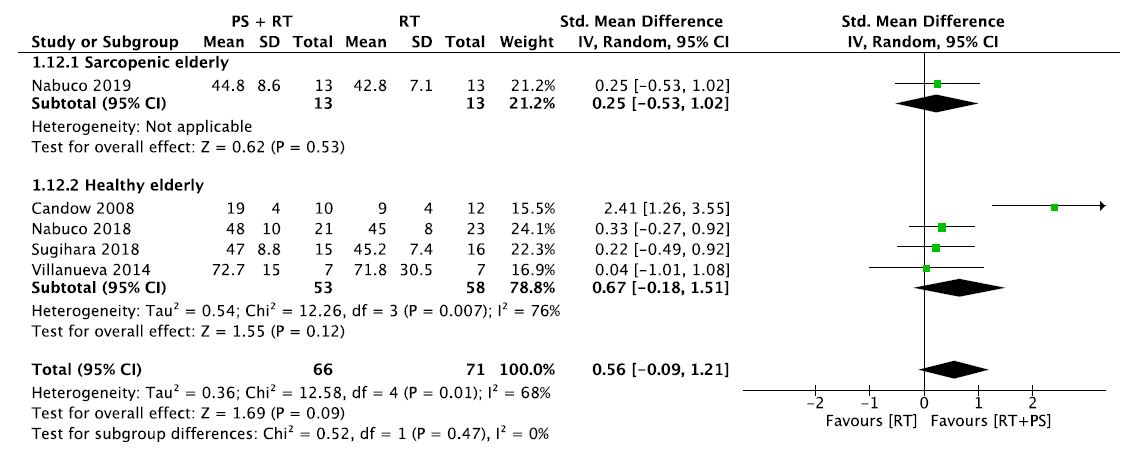


**Figure 2:** EESS Strength by Sarcopenia Condition


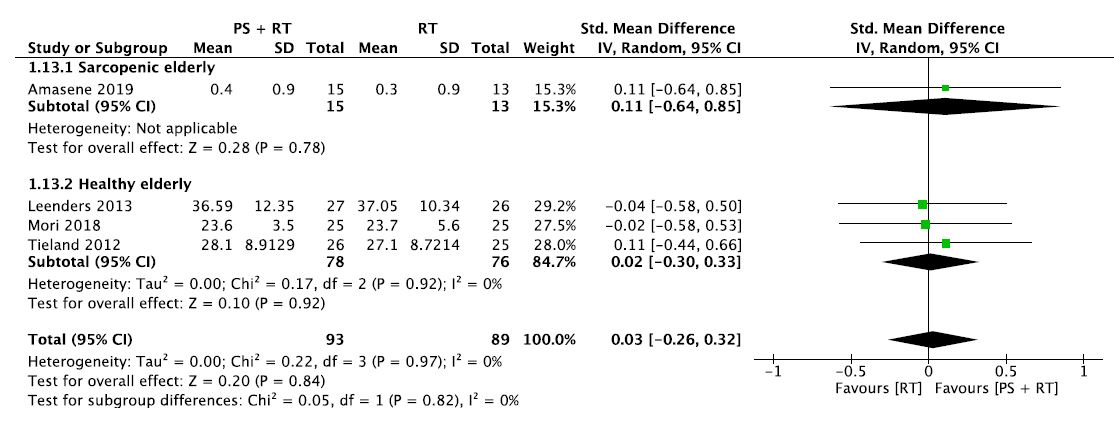


**Figure 3:** Handgrip by Sarcopenia Condition


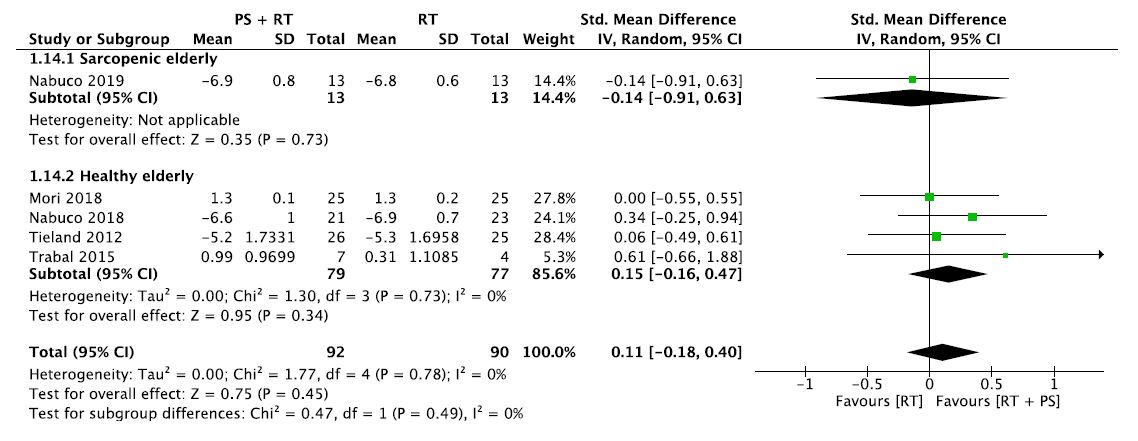


**Figure 4:** Gait Speed by Sarcopenia Condition


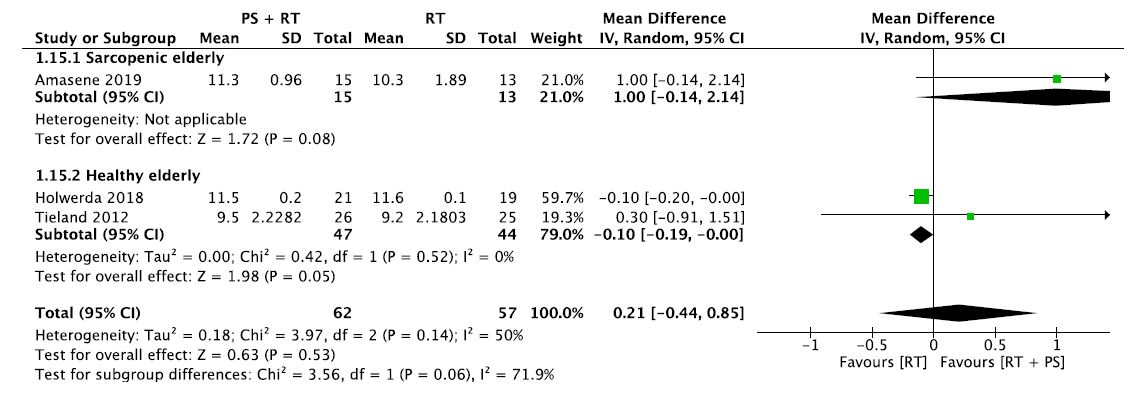


**Figure 5:** SPPB by Sarcopenia Condition


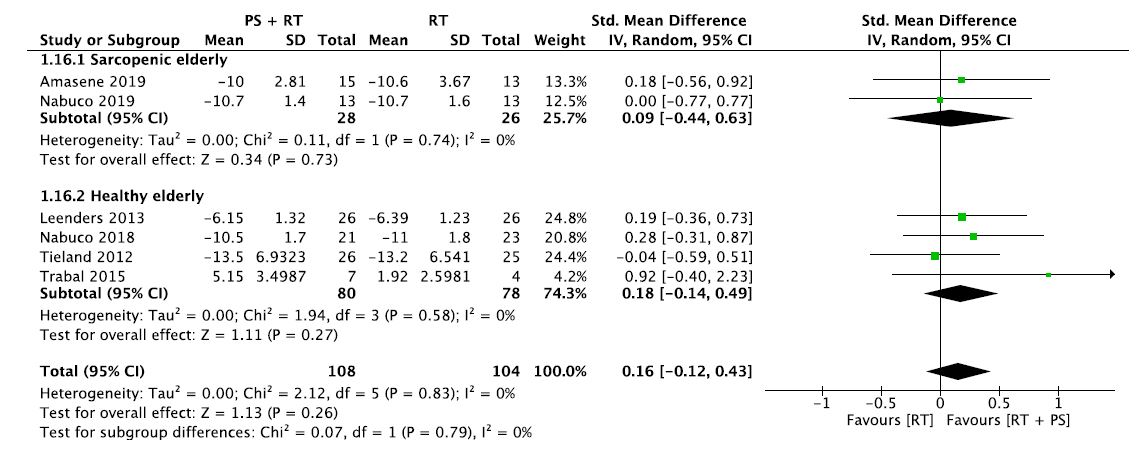


**Figure 6:** 5 Chair Raise by Sarcopenia Condition
