# Supplementary material for: Effectiveness of Protein Supplementation Combined with Resistance Training on Muscle Strength and Physical Performance in Elderly: A Systematic Review and Meta-Analysis
Source: Nutrients. 2020 Aug 27;12(9):2607. doi: 10.3390/nu12092607 (PMC7551830; doi:10.3390/nu12092607)
Supplement: Supplementary file 1 [file nutrients-12-02607-s001.zip › supplementary/Supplementary S5. Complete sensitivity Analysis evaluating heterogeneity across studies.docx]

**Supplementary S5:** Complete sensitivity Analysis evaluating heterogeneity across studies.

| **Lower Limb Strength** | | | |
| --- | --- | --- | --- |
| Study dropped out | SMD | 95% CI | I^2^ |
| Arnason 2013 | -0.01 | [-0.22, 0.20] | 17% |
| Candow 2008 | 0.01 | [-0.18, 0.20] | 16% |
| Holwerda 2018 | 0.05 | [-0.12, 0.22] | 0% |
| Leenders 2013 | 0.01 | [-0.19, 0.20] | 17% |
| Mori 2018 | 0.03 | [-0.16, 0.21] | 11% |
| Nabuco 2018 | -0.02 | [-0.21, 0.17] | 13% |
| Nabuco 2019 | 0.01 | [-0.18, 0.20] | 17% |
| Stragier 2016 | -0.00 | [-0.19, 0.19] | 17% |
| Sugihara 2018 | -0.01 | [-0.20, 0.18] | 16% |
| Tieland 2012 | 0.02 | [-0.18, 0.21] | 16% |
| Trabal 2015 | -0.02 | [-0.18, 0.14] | 0% |
| Verdijk 2009 | -0.04 | [-0.20, 0.13] | 0% |
| Villanueva 2014 | -0.01 | [-0.19, 0.18] | 16% |
| Zdzieblik 2015 | -0.00 | [-0.20, 0.19] | 17% |
| None | -0.00 | [-0.18, 0.18] | 11% |
| SMD Standard Mean Difference; CI Confidence Interval; I^2^ Heterogeneity Statistic | | | |

| **Upper Limb Strength** | | | |
| --- | --- | --- | --- |
| Study dropped out | SMD | 95% CI | I^2^ |
| Candow 2008 | 0.24 | [-0.12, 0.61] | 0% |
| Nabuco 2018 | 0.66 | [-0.25, 1.57] | 76% |
| Nabuco 2019 | 0.67 | [-0.18, 1.51] | 76% |
| Sugihara 2018 | 0.68 | [-0.18, 1,54] | 75% |
| Villanueva 2014 | 0.68 | [-0.09, 1.45] | 75% |
| None | 0.56 | [-0.09, 1.21] | 68% |
| SMD Standard Mean Difference; CI Confidence Interval; I^2^ Heterogeneity Statistic | | | |

| **Handgrip** | | | |
| --- | --- | --- | --- |
| Study dropped out | SMD | 95% CI | I^2^ |
| Amasene 2019 | 0.02 | [-0.30, 0.33] | 0% |
| Leenders 2013 | 0.06 | [-0.29, 0.40] | 0% |
| Mori 2018 | 0.05 | [-0.29, 0.39] | 0% |
| Tieland 2012 | -0.00 | [-0.34, 0.34] | 0% |
| None | 0.03 | [-0.26, 0.32] | 0% |
| SMD Standard Mean Difference; CI Confidence Interval; I^2^ Heterogeneity Statistic | | | |

| **Gait Speed** | | | |
| --- | --- | --- | --- |
| Study dropped out | SMD | 95% CI | I^2^ |
| Mori 2018 | 0.15 | [-0.19, 0.50] | 0% |
| Nabuco 2018 | 0.04 | [-0.30, 0.37] | 0% |
| Nabuco 2019 | 0.15 | [-0.16, 0.47] | 0% |
| Tieland 2012 | 0.13 | [-0.21, 0.48] | 0% |
| Trabal 2015 | 0.08 | [-0.22, 0.38] | 0% |
| None | 0.11 | [-0.18, 0.40] | 0% |
| SMD Standard Mean Difference; CI Confidence Interval; I^2^ Heterogeneity Statistic | | | |

| **SPPB** | | | |
| --- | --- | --- | --- |
| Study dropped out | SMD | 95% CI | I^2^ |
| Amasene 2019 | -0.10 | [-0.19, -0.00] | 0% |
| Holwerda 2018 | 0.67 | [-0.16, 1.50] | 0% |
| Tieland 2012 | 0.30 | [-0.74, 1.33] | 72% |
| None | 0.21 | [-0.44, 0.85] | 50% |
| SMD Standard Mean Difference; CI Confidence Interval; I^2^ Heterogeneity Statistic | | | |

| **5 Chair Raise** | | | |
| --- | --- | --- | --- |
| Study dropped out | SMD | 95% CI | I^2^ |
| Amasene 2019 | 0.15 | [-0.14, 0.44] | 0% |
| Leenders 2013 | 0.15 | [-0.17, 0.46] | 0% |
| Nabuco 2018 | 0.12 | [-0.18, 0.43] | 0% |
| Nabuco 2019 | 0.18 | [-0.11, 0.47] | 0% |
| Tieland 2012 | 0.22 | [-0.09, 0.53] | 0% |
| Trabal 2015 | 0.12 | [-0.15, 0.40] | 0% |
| None | 0.16 | [-0.12, 0.43] | 0% |
| SMD Standard Mean Difference; CI Confidence Interval; I^2^ Heterogeneity Statistic | | | |
